# Novel Reassortant Avian Influenza A(H3N8) Virus Isolated from a Wild Bird in Jiangxi, China

**DOI:** 10.1128/MRA.01163-19

**Published:** 2019-11-14

**Authors:** Ruiyun Li, Tao Zhang, Jian Xu, Jianyu Chang, Bing Xu

**Affiliations:** a MRC Centre for Global Infectious Disease Analysis, Department of Infectious Disease Epidemiology, School of Public Health, Faculty of Medicine, Imperial College London, London, United Kingdom; b Ministry of Education Key Laboratory for Earth System Modelling, Department of Earth System Science, Tsinghua University, Beijing, China; c Centre for Healthy Cities, Institute for China Sustainable Urbanization, Tsinghua University, Beijing, China; d School of Geography and Environmental Science, Ministry of Education Key Laboratory of Poyang Lake Wetland and Watershed Research, Jiangxi Normal University, Nanchang, Jiangxi, China; e College of Veterinary Medicine, China Agricultural University, Beijing, China; DOE Joint Genome Institute

## Abstract

Here, we report the detection of a reassortant avian influenza A(H3N8) virus isolated from a wild bird in Poyang Lake, Jiangxi, China, in 2014. Phylogenetic analyses indicated that this virus is most likely derived from the Eurasian-origin H3Ny and HxN8 viruses and two strains endemic to China, namely, H5N1 and H5N6.

## ANNOUNCEMENT

Avian influenza virus (AIV) is a single-stranded segmented negative-sense RNA virus which belongs to the *Alphainfluenzavirus* genus in the family *Orthomyxoviridae* (1). Previous surveillance conducted in live bird markets and farms has demonstrated the cocirculation of multiple AIV subtypes in domestic poultry in China (2, 3). Among these subtypes, the H3 viruses are one of the most prevalent subtypes in domestic ducks and have undergone extensive reassortment events (3, 4). Notably, H3 viruses have reassorted with subtypes endemic to China, such as H5N6 (2, 5), indicating a potential risk to public health. However, knowledge about the complexity of H3 evolution in the wild bird population is still limited.

To gain insight into viral evolution in wild bird reservoirs, we undertook routine bird surveillance in Jiangxi Province, China, from 2014 to 2016. Here, we provide a report on the identification and sequence analysis of a novel reassortant H3N8 virus isolated from a wild bird in Jiangxi, China, in 2014. The complete details of the surveillance and viral isolation were presented in our previous study (6). In brief, 13 out of 488 tracheal and cloacal swab samples collected had hemagglutination activity. The viral RNAs of these positive samples were extracted from allantoic fluid using the RNeasy mini kit (Qiagen, Hilden, Germany). The reverse transcription was subsequently carried out using the SuperScript III reverse transcriptase (RT) PCR kit (Invitrogen, USA), and subtype was determined using PCR of a marker gene (7, 8). One sample isolated from a mallard duck was identified as H3N8 and designated A/mallard/Jiangxi/G98/2014(H3N8). Each segment of this sample was amplified with a Phusion high-fidelity PCR system (New England BioLabs, Ipswich, MA, USA), adhering to Hoffmann’s approach (9), and sequenced as individual amplicons using the Applied Biosystems automated 3730xl DNA analyzer. For each segment, only the coding region was sequenced, which is base pairs 1 to 2280 (PB2), base pairs 1 to 2274 (PB1), base pairs 1 to 2151 (PA), base pairs 1 to 1701 (HA), base pairs 1 to 1497 (NP), base pairs 1 to 1413 (NA), base pairs 1 to 982 (M), and base pairs 1 to 844 (NS). The G+C contents were calculated using DNAStar v7.1.0 and were 44.56% (PB2), 43.38% (PB1), 43.93% (PA), 44.50% (HA), 47.23% (NP), 45.58% (NA), 47.21% (M), and 44.46% (NS).

**FIG 1:**
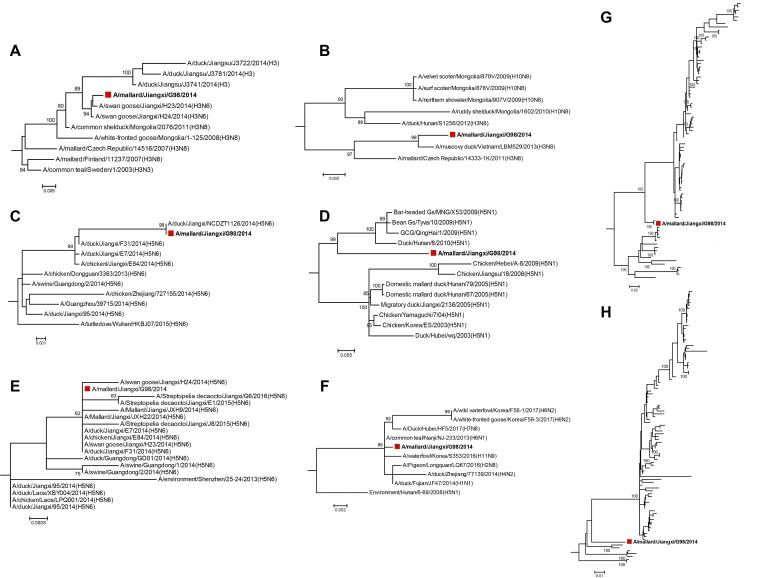
Phylogeny of all gene segments of Jiangxi H3N8 virus. Phylogeny of the HA (A), NA (B), PB1 (C), PA (D), M (E), NS (F), PB2 (G), and NP (H) genes was inferred using the maximum likelihood method with 1,000 bootstrap replicates in MEGA v6.06. The Kimura 2-parameter substitution model was selected with the assumption of a gamma distribution with invariant rates among sites. The Jiangxi H3N8 virus is highlighted by the red square.

Phylogenetic analyses suggested that all gene segments of this Jiangxi H3N8 virus belonged to the Eurasian lineage (Fig. 1). The HA and NA genes are likely evolved from multiple H3Ny (N3, N6, and N8) and HxN8 (H3 and H10) subtypes. Phylogeny of internal genes showed that the Jiangxi H3N8 virus is capable of reassorting with strains endemic to China, i.e., an H5N6-origin reassortment for the PB1 and M genes and an H5N1-origin reassortment for the PA and NS genes. The origins for the PB2 and NP genes were unknown.

A BLAST search in the GenBank database demonstrated a high nucleotide identity of the H3N8 virus with multiple subtypes circulating in wild and domestic birds in Asian countries (Table 1). The H3N8 virus contains the HA gene closest to that of A/common shelduck/Mongolia/2076/2011(H3N8), with 98.48% identity, whereas its NA genes were closely related to A/duck/Mongolia/123/2014(H10N8) and A/muscovy duck/Vietnam/LBM529/2013(H3N8), with an identity of 98.74%. This genetic relationship indicates that the surface genes of H3N8 virus might have been introduced from Mongolia and Vietnam through long-term migration of wild birds. Internal genes of the Jiangxi H3N8 virus were identified to share the closest relationship with viruses recently isolated from China. Of note, the PB2, PB1, and M genes of the H3N8 virus were most closely related to an H5N6 isolate endemic to China, i.e., A/chicken/Jiangxi/NCDZT1126/2014(H5N6), with identities of 93.60%, 100%, and 99.51%, respectively. The PA and NP genes shared highest identity (>98%) with A/wild bird/Jiangxi/P419/2016(H6N8). The NS gene shared 99.88% identity with the closest A/common teal/Nanji/NJ-233/2013(H6N1) strain.

**TABLE 1:** Nucleotide sequence identities between the Jiangxi H3N8 virus and the closest homologs in the GenBank database

Gene	Virus	GenBank accession no.	Subtype	Identity (%)
HA	A/common shelduck/Mongolia/2076/2011	KF501077	H3N8	98.47
NA	A/duck/Mongolia/123/2014	MK978914	H10N8	98.74
	A/muscovy duck/Vietnam/LBM529/2013	AB916669	H3N8	98.74
PB2	A/chicken/Jiangxi/NCDZT1126/2014	KP090444	H5N6	93.60
PB1	A/chicken/Jiangxi/NCDZT1126/2014	KP090445	H5N6	100.00
PA	A/wild bird/Jiangxi/P419/2016	KX867856	H6N8	98.43
NP	A/wild bird/Jiangxi/P419/2016	KX867858	H6N8	98.02
M	A/chicken/Jiangxi/NCDZT1126/2014	KP090450	H5N6	99.51
NS	A/common teal/Nanji/NJ-233/2013	KJ907667	H6N1	99.88

The detection of a novel reassortant H3N8 virus is suggestive of the complex reassortment of multiple AIV subtypes in the wild bird population. Our study provides evidence to enhance the surveillance of avian influenza viruses and to further understand their reassortment and diversity in wild birds in Poyang Lake.

### Data availability.

The genome sequences of the Jiangxi H3N8 virus have been deposited in the GenBank database under the accession numbers MN473210 to MN473217.

## References

[1] Lefkowitz EJ, Dempsey DM, Hendrickson RC, Orton RJ, Siddell SG, Smith DB 2018 Virus taxonomy: the database of the International Committee on Taxonomy of Viruses (ICTV). Nucleic Acids Res 46:D708–D717. doi:10.1093/nar/gkx932. 29040670 PMC5753373

[2] Li X, Yang J, Liu B, Jia Y, Guo J, Gao X, Weng S, Yang M, Wang L, Wang L, Cui J, Chen H, Zhu Q 2016 Co-circulation of H5N6, H3N2, H3N8, and emergence of novel reassortant H3N6 in a local community in Hunan Province in China. Sci Rep 6:25549. doi:10.1038/srep25549. 27151540 PMC4858758

[3] Deng G, Tan D, Shi J, Cui P, Jiang Y, Liu L, Tian G, Kawaoka Y, Li C, Chen H 2013 Complex reassortment of multiple subtypes of avian influenza viruses in domestic ducks at the Dongting Lake region of China. J Virol 87:9452–9462. doi:10.1128/JVI.00776-13. 23804642 PMC3754128

[4] Luo S, Xie Z, Xie Z, Xie L, Huang L, Huang J, Deng X, Zeng T, Wang S, Zhang Y, Liu J 2017 Surveillance of live poultry markets for low pathogenic avian influenza viruses in Guangxi Province, southern China, from 2012–2015. Sci Rep 7:17577. doi:10.1038/s41598-017-17740-0. 29242521 PMC5730573

[5] Liu T, Xie Z, Luo S, Xie L, Deng X, Xie Z, Huang L, Huang J, Zhang Y, Zeng T, Wang S 2015 Characterization of the whole-genome sequence of an H3N6 avian influenza virus, isolated from a domestic duck in Guangxi, southern China. Genome Announc 3:e01190-15. doi:10.1128/genomeA.01190-15. 26472834 PMC4611686

[6] Zhang T, Li R, Zhu G, Chang J, Xu B 2019 First detection of a novel reassortant avian influenza A(H5N6) clade 2.3.2.1c virus, isolated from a wild bird in China. Microbiol Resour Announc 8:e00797-19. doi:10.1128/MRA.00797-19. 31488532 PMC6728642

[7] Tsukamoto K, Ashizawa T, Nakanishi K, Kaji N, Suzuki K, Shishido M, Okamatsu M, Mase M 2009 Use of reverse transcriptase PCR to subtype N1 to N9 neuraminidase genes of avian influenza viruses. J Clin Microbiol 47:2301–2303. doi:10.1128/JCM.02366-08. 19403772 PMC2708491

[8] Lee MS, Chang PC, Shien JH, Cheng MC, Shieh HK 2001 Identification and subtyping of avian influenza viruses by reverse transcription-PCR. J Virol Methods 97:13–22. doi:10.1016/S0166-0934(01)00301-9. 11483213

[9] Hoffmann E, Stech J, Guan Y, Webster RG, Perez DR 2001 Universal primer set for the full-length amplification of all influenza A viruses. Arch Virol 146:2275–2289. doi:10.1007/s007050170002. 11811679

